# A Proposed British Nursing Association

**Published:** 1887-12-03

**Authors:** 


### Proposed "British Nursing Association."

We are informed that Mrs. Fenwick, with the co-operation and help of Miss Wood, has invited certain lady superintendents and matrons to a meeting to consider the desirability of establishing one more institution with the above title.

This new scheme is stated to aim at establishing a separate association for nurses, *i.e.*, a British Nursing Association, with the following objects, amongst others: To build homes for convalescent nurses and for the aged; to provide annuities, pensions, and sick relief; to confer degrees, diplomas, and certificates upon nurses; to establish a new journal; to procure a Charter of Incorporation; and generally to take in hand numerous projects, which will collectively require many thousands of pounds to work. We have been requested to publish the following letter from Mrs. Bluett, who is deservedly regarded as one of the most practical and capable members of the nursing profession. The letter is written, we believe, in reply to one sent by Mrs. Fenwick asking Mrs. Bluett to join the council of the proposed association:—

"Fitzroy House, 16 and 17, Fitzroy-square, W.,
"November 29th, 1887.

"Dear Mrs. Fenwick,—Many thanks for your letters, which I have carefully considered. I have taken an opportunity of conferring with those who are entitled to speak with authority in the nursing world, and their opinion is decidedly against the probability of any successful working of what you propose. The experience we have so lately had in connexion with the Sectional Committee on Nursing and Domestic Management convinces me that ladies' committees are altogether a mistake; others must be of the same opinion, for I believe nine members have retired because no work was being done! I am also persuaded that 'The Hospitals Association' (the council of which consists of representatives of all classes of institutions, and all sections of hospital work) is the proper medium of communication with the nursing world, and to act wisely, and insure the confidence of the public they will place the registration scheme under a joint committee of gentlemen and of ladies, who are themselves fully trained and certificated nurses. I would remind you that several of the proposed objects on your printed slips have been already reduced to practical form by 'The Hospitals Association,' which has now placed them upon a sound basis. The remainder are mainly those which I myself proposed for the consideration of the 'Sectional Committee on Nursing,' and these are now submitted to the committee with the view of practical fulfilment so far as may be found possible. It may sometimes be justifiable, and even necessary, to establish a new organisation, with new objects which cannot otherwise be obtained; but I am always opposed to the unnecessary multiplication of associations, of which we have far too many, especially when, as in this instance, all the important objects are already covered by existing institutions. To be quite frank, I feel that a 'British Nurses' Association,' managed as you propose, would not be calculated to succeed; and I am sure that we women ought not to incur a financial responsibility which must amount to many thousands of pounds. I at any rate feel that 'The Hospitals Association,' by affording a wider and firmer basis, can give more assistance and help in promoting the objects on your paper, than are likely to be obtained from any new organisation whatever; and for this and other reasons I have stated, I must ask you to excuse me from taking any part in this new movement, or having my name mixed up with the same. "C. R. Bluett."

We shall be glad to know how far the opinions Mrs. Bluett has so ably expressed are those of the members of the nursing profession, who, by virtue of their holding certificates, are entitled to speak with authority.

